# Intellectual Brilliance and Presidential Performance: Why Pure Intelligence (or Openness) Doesn’t Suffice

**DOI:** 10.3390/jintelligence6020018

**Published:** 2018-03-23

**Authors:** Dean Keith Simonton

**Affiliations:** Department of Psychology, University of California, Davis, CA 95616, USA; dksimonton@ucdavis.edu

**Keywords:** presidential performance, Intellectual Brilliance, Openness to Experience, IQ

## Abstract

In recent years it has become popular on the internet to debate the IQ of the incumbent president of the United States. Yet, these controversies (and hoaxes) presume that IQ has some relevance to understanding the president’s actual performance as the nation’s leader. This assumption is examined by reviewing the empirical research on the intelligence–performance association in political leadership, with a special focus on U.S. presidents. The review starts by discussing at-a-distance assessment techniques, a method that has yielded reliable and valid measures of IQ, Intellectual Brilliance, and Openness to Experience; three correlated even if separable concepts. The discussion then turns to the reliable and valid measurement of presidential performance—or “greatness”—via successive surveys of hundreds of experts. These two lines of research then converged on the emergence of a six-predictor equation, in which Intellectual Brilliance plays a major role, to the exclusion of both IQ and Openness. The greatest presidents are those who feature wide interests, and who are artistic, inventive, curious, intelligent, sophisticated, complicated, insightful, wise, and idealistic (but who are far from being either dull or commonplace). These are the personal traits we should look for in the person who occupies the nation’s highest office if we seek someone most likely to solve the urgent problems of today and tomorrow.

## 1. Introduction

This symposium of thought pieces is dedicated to the following question: “If intelligence is truly important to real-world adaptation, and IQs have risen 30+ points in the past century (Flynn effect), then why are there so many unresolved and dramatic problems in the world, and what can be done about it?” Needless to say, this issue has many possible responses. For instance, it just may be the case that while IQs have grown at, say, a roughly linear pace (even ignoring any asymptotic leveling off), the problems faced by the world have grown exponentially. Indeed, as evinced by the infamous “hockey stick graph” that Al Gore used in his 2006 documentary, “An Inconvenient Truth”, to argue for human-caused global warming, problems could actually accelerate faster than exponentially, leaving the Flynn effect in the dust. 

That said, I would like to treat a different answer here. Very often, scientists and other experts demonstrate the wherewithal to arrive at effective solutions to world problems, but they are powerless to implement those solutions because the power of implementation is confined to the highest levels of leadership, such as a president, prime minister, or even dictator. If intelligence is required for a leader to effectively understand and enact the appropriate measures, then perhaps the intelligence of the population (or voters) is not really relevant. What really matters is the intellect of the heads of state. In other words, the Flynn effect is not really relevant. What is critical is the particular kind of intellect found in the world’s leaders. Here, I would like to concentrate on a specific manifestation: the relation between intelligence and performance in the United States presidency. In particular, I will review the relevant results from a research program addressing this question that began in 1986, and eventually came to include all U.S. presidents between George Washington and George W. Bush [1].^1^

As some readers may remember, during the 2016 presidential campaign, a major candidate, Donald Trump, explicitly boasted that he was extremely smart. Indeed, after his inauguration in early 2017, he made the more specific claim that he enjoyed a superlative IQ, and even challenged his own Secretary of State, Rex Tillerson, to take an IQ test. Moreover, the president eventually asserted that his intelligence was not just very high, but even attained the elevated level of genuine genius. Unfortunately, because I have been conducting empirical inquiries into presidential intelligence since the 1980s [2], I found myself dragged into the ensuing internet debate over Trump’s actual IQ. Was it really true that he has an IQ of 156; well over three standard deviations above the population mean? Nor was this the first time I was put in the uncomfortable position of publically arbitrating the supposed IQs of U.S. chief executives. Back in 2001 a widely circulated internet hoax maintained that George W. Bush’s intelligence was well below average, weighing in at a mere IQ of 91. When I published a far more reasonable IQ estimate five years later [3], I found myself under vicious attacks by those who argued that my estimate was way too high—or still way too low. Not understanding the statistical methods that I had used, the common assumption was that my endeavors were purely partisan. In any event, it seems that many people out there really care about the IQ scores of the White House incumbent. Just google “presidents IQ” for the evidence. 

But why should it even matter in the first place? Presumably, the answer is that general intelligence, as assessed by IQ tests, bears a strong positive association with actual presidential performance. Presidents with higher IQs would supposedly do a better job solving the nation’s problems as well as the problems confronting the world at large, whether those problems concern military conflicts, terrorism, immigration, poverty, crime, discrimination, or climate change. But what scientific evidence do we actually possess for such a relationship? After all, few if any U.S. presidents can claim a certified score on an IQ test, and the vast majority of presidents died before taking such a test was even possible. Furthermore, how is it conceivable to assess a president’s overall leadership? Without such an assessment, the intelligence–performance question becomes moot anyway. 

Here, I begin by addressing the assessment of intelligence and related constructs, and then turn to the measurement of leader performance. I conclude by discussing their correspondence. It will then become apparent that intelligence, as usually defined, is a far too narrow a construct to provide an optimal predictor of presidential leadership. 

## 2. Intellect, IQ, Intellectual Brilliance, and Experiential Openness

The only way to assess intelligence in all of the U.S. presidents is to utilize some at-a-distance assessment technique [4,5]. An early prototype was Woods’ [6] attempt to estimate Intellect in members of European royal families by means of the personality descriptors routinely supplied in biographical entries. Woods’ method was subsequently applied by Cox [7], Thorndike [8,9], and later still, by Simonton [10], who focused on just those royals who assumed their nation’s throne as a monarch (king, queen, or sultan). Simonton’s study showed that Woods’ ratings of Intellect were highly correlated with such biographical descriptors as Intelligent, Able, Shrewd, and Educated. One primary drawback of these calculations is that the evaluators were aware of the identity of those being rated, a deficiency that has a remedy, as will be seen shortly. 

An alternative approach was introduced by Cox [7] as part of Terman’s [11] classic longitudinal study of over 1500 high-IQ children (for background, see [12,13]). Employing the original definition of IQ as the literal quotient of mental age divided by chronological age multiplied by 100, Cox calculated estimates for 301 geniuses, including both creators and leaders. The IQ estimates were based on detailed chronologies of early intellectual development, using multiple independent raters to obtain reliable scores. In essence, the intelligence measure is a gauge of precocity in intellectual development during childhood, adolescence, and early adulthood (see also [14] for a slight variation on this technique). An especially impressive feature of her estimation strategy is that she provided four estimates rather than just one. She first calculated separate IQs for ages 0–16 and 17–26, and then provided both a raw estimate and an estimate corrected for data reliability (which was made possible by her using multiple independent raters). Unhappily, only 8 of the 301 had served as the U.S. chief executive, and all of these were active prior to the 20th century (viz. George Washington, John Adams, Thomas Jefferson, James Madison, J. Q. Adams, Andrew Jackson, Abraham Lincoln, and U. S. Grant). Not a large sample of presidents, to be sure. 

Yet another method returns to biographical descriptors, this time applied to presidents of the United States, rather than European monarchs [2]. Moreover, important improvements were implemented over what was conducted by Woods [6], Thorndike [8,9], and Simonton [10]. To begin with, a research team extracted the descriptors from biographical materials with all identifying information removed. To avoid the introduction of potential political biases, these materials were carefully selected to represent the consensus of historical scholarship on the American presidency [15]. Next, a totally separate team of several independent raters used these anonymous extracts to check off the applicable descriptors using the Gough Adjective Check List (ACL) [16].^2^ Because not all 300 adjectives could be reliably assessed, the descriptors were reduced to a subset of 110 assessments that had sufficiently high reliability coefficients. These reliable assessments were then subjected to a factor analysis that yielded 14 orthogonal dimensions. For our present purposes, just one of these factors is the most important; namely, the one labeled Intellectual Brilliance. This factor had salient positive loadings on Wide Interests (0.85), Artistic (0.84), Inventive (0.76), Curious (0.74), Intelligent (0.64), Sophisticated (0.62), Complicated (0.61), Insightful (0.54), Wise (0.46), and Idealistic (0.43), but negative loadings on Dull (−0.71) and Commonplace (−0.41). The reliability (coefficient alpha) for Intellectual Brilliance was 0.90, a highly respectable figure [2]. Better yet, scores on this measure were obtained for all 39 U.S. presidents between Washington and Ronald Reagan, inclusively. Notably, the factor scores were validated by calculating correlations with independent assessments of related constructs, such as IQ, creativity, charisma, idealism, book authorship, and birth order (all positive except the last; [2,17]; cf. [7,9,20,21,22]). Critically, Intellectual Brilliance does *not* correlate with the president’s party affiliation, thus indicating a lack of bias in the biographical reference works [3]. 

Admittedly, exploratory factor analysis is sometimes more an art than a science. Although the rotated factors exhibited the desired simple structure, assigning labels to the extracted factors can always be subjected to second-guessing. That possibility certainly applies to the cluster of traits that were styled as Intellectual Brilliance. At that time, and in the numerous subsequent studies published since, that label was deliberately chosen to make intelligence an adjective rather than a noun. The configuration of descriptors were taken to indicate a certain pervasive brilliance of an intellectual kind. More specifically, Intellectual Brilliance can be defined as an inclusive cognitive propensity that spans broad and artistic interests, a pronounced curiosity and inventiveness, noticeable sophistication and insightfulness, plus more than average wisdom and idealism. This cognitive emphasis contrasted with the other factors, which more often concerned emotions, motivations, or attitudes (e.g., Forcefulness, which was defined by the descriptors of Energetic, Active, Determined, Demanding, and Restless).^3^

Just as importantly, Intellectual Brilliance obviously encompasses far more than mere intelligence. First of all, the descriptor Intelligent cannot be taken to define the factor, because its loading falls right in the middle of the pack rather than having the highest loading of all (viz. 0.21 below Interests Wide, but 0.21 above Idealistic). Moreover, most of the component descriptors concern creativity, and to a lesser extent, sophistication, wisdom, and idealism (cf. [25]). Furthermore, many adjectives seem to tap into facets of the Openness to Experience dimension of the Big Five Personality Model [26]. This factor includes the six facets of “Openness to Fantasy, Aesthetics, Feelings, Actions, Ideas, and Values” [27] (p. 223). In fact, the following ACL adjectives correlate positively with Openness scores in the general population: Wide Interests, Imaginative, Intelligent, Original, Insightful, Curious, Sophisticated, Artistic, Clever, Inventive, Sharp-Witted, Ingenious, and Wise (but with negative correlations for Commonplace, Narrow Interests, Simple, Shallow, and Unintelligent; [26]). The overlap with Intellectual Brilliance is obvious. This conspicuous overlap also suggests that Intellectual Brilliance might only be serving as a proxy for Openness to Experience. Such a connection is significant insofar as Openness has already been shown to have a strong association with both creativity and leadership [27,28]. So perhaps we should really speak of Openness rather than Intellectual Brilliance—and thus drop intellect or intelligence out of the discussion altogether.

Fortunately, this possibility can be directly addressed by using yet another at-a-distance method for assessing individual differences in historical figures [5]. Because eminent personalities usually attract the attention of biographers, their biographers become ready-made experts regarding the personal qualities of their subjects. As such, the biographers can respond to surveys using observer-based rather than self-report measures, including versions of Big Five personality questionnaires. This technique was actually applied to presidents of the United States, most of whom can claim multiple biographies [29,30]; for methodological critique, see [31]. The use of multiple biographers as survey respondents would thus help reduce the intrusion of partisan bias. The upshot of this was that 32 presidents eventually received assessments on all five factors, including Openness to Experience. Not surprisingly, these observer-based Openness scores correlate highly with the Intellectual Brilliance scores (*r* = 0.69; [3]). Yet, it is also the case that both of these scores correlate positively with Cox’s [7] IQ estimates, predicated on a totally distinct method (*r*s = 0.70−0.92, depending on the specific Cox estimates chosen [3]). Perhaps these high correlations should come as no surprise given that “intelligence” and related traits are included in both assessments. But the real question is which of the three assessments most strongly correlates with actual presidential performance: IQ, Intellectual Brilliance, or Openness to Experience? Once one of the three is accounted for, do the remaining two become superfluous? To address this issue, we first must discuss how that performance is assessed. 

## 3. Presidential Leadership, Performance, and Greatness

Experts on the U.S. presidents, largely historians of American political history, have been repeatedly surveyed to determine the performance of former presidents (ignoring incumbents who have not yet completed their terms). Indeed, the first such survey was published in 1948 [32]. Although the ratings will change somewhat from survey to survey (not even counting the addition of new former presidents), the shifts tend to be relatively modest, comparable to the shifts in relative individual rankings in test–retest reliabilities for IQ scores. As a direct consequence, research shows that the alternative assessments exhibit a substantial consensus [33]. Presidents like Lincoln, Washington, and Franklin Roosevelt are placed towards the top, while presidents like Andrew Johnson, Warren Harding, and Richard Nixon regularly fall towards the bottom. Plus, the more mediocre chief executives will compete in the middle two quartiles (or roughly ranks 10 to 30). So strong is the agreement that when a measure of presidential performance is defined based on a factor analysis, the resulting 12-item composite features a (coefficient alpha) reliability of 0.99, which is as good as it possibly gets [3]. This reliable composite has frequently been labeled a presidential “greatness” factor [33,34,35,36].

From time to time, survey respondents will be asked to provide more finely differentiated evaluations of presidential performance [21,37]. The ratings then allow us to pinpoint the more specific leadership qualities that are getting at least partially tapped by the global measure; much like the correlations between general intelligence (Spearman’s *g*) and measures of separate cognitive abilities, whether verbal, visual, spatial, mathematical, of memory, etc. Thus, we find that the comprehensive greatness assessment agrees with Maranell’s survey ([21]; of 571 experts) results for Accomplishments (*r* = 0.97), Strength (*r* = 0.96), Presidential Prestige (*r* = 0.95), and Activity (*r* = 0.90), and with Ridings and McIver’s survey ([37]; of 719 experts) findings for Accomplishments (*r* = 0.94), Presidential Leadership (*r* = 0.93), Appointments (*r* = 0.90), and Political Skill (*r* = 0.90). It should be obvious from the high correlations that when these separate assessments are factor-analyzed along with the overall assessments, a single-factor solution is still obtained (see also [38]). 

One might be tempted to ascribe the above agreement to some grand halo effect in the evaluation of presidential leadership. Yet, opposed to this attribution is the fact that some hypothesized performance criteria do not correspond strongly with the broad greatness factor. For example, Ridings and McIver [37] also had expert respondents gauge the presidents on Character and Integrity, but this measure did not correlate very highly with the other assessments [39]. It is sad to say, but these virtues do not seem to define core components of presidential greatness! Some great presidents are not great persons, just as some great persons do not make great presidents (e.g., Woodrow Wilson versus Jimmy Carter). Any composite indicator is better left without Character and Integrity included. 

## 4. Intelligence–Performance Correspondence

Using a sample of 342 European monarchs, Simonton [10,40] conducted the first assessment of the association between intelligence and leader performance. Rated intelligence (an extension of Woods’ [6] “Intellect” measure) exhibited statistically significant correlations with a monarch’s Leadership (*r* = 0.67), achieved Eminence (*r* = 0.32), assessed Morality (*r* = 0.23, an extension of Woods’ [6] “Virtue” measure), and objective Historical Activity (*r* = 0.13, a measure of the number of notable events that occurred during his or her reign). Although promising, these two inquiries suffered from the problem mentioned earlier: the measures were not generated by raters blind to the identity of the leaders being rated. In addition, because monarchs represent a rather outdated form of political leadership in the 20th century, it seemed desirable to look at a political position of unquestioned contemporary importance: the presidents of the United States. 

Such was the impetus for the investigation mentioned earlier that obtained an Intellectual Brilliance assessment for the first 39 U.S. presidents [2]. For the primary performance criterion, Simonton used the most recent global assessment, a survey of 846 experts on the presidency [41], finding a sizable correlation (*r* = 0.56). In fact, Intellectual Brilliance was the only factor out of the 14 extracted dimensions—such as Moderation, Machiavellianism, Achievement Drive, Inflexibility, and Conservatism—that exhibited any association whatsoever with presidential greatness. This is not to say that these other traits are utterly irrelevant, but only that their repercussions tend to be confined to particular leader behaviors, and frequently their effects are moderated by situational variables [1,2]. For example, the magnitude of Inflexibility influences the president’s use of the legislative veto power, but only in interaction with the size of the chief executive’s electoral mandate and the degree to which his party controls Congress [42]. In a nutshell, Inflexibility only proves adaptive when the president has strong political support among voters and legislators. 

As a further confirmation of its predictive power, Intellectual Brilliance was shown to correlate significantly with all prior survey results, essentially yielding a rare seven-fold replication. Finally, because several other variables have been shown to predict presidential performance besides Intellectual Brilliance ([22,33]; see also [1]), Simonton [2] examined whether this single dispositional factor would survive as a predictor when placed in a multiple regression equation that included total years in office, years as wartime commander-in-chief, assassination, status as a war hero, and administration scandals (a negative predictor). Intellectual Brilliance retained its predictive utility (standardized partial regression coefficient β = 0.26). The six-predictor equation accounted for 82% of the variance in assessed performance, a result that has been replicated several times as new former presidents are added to the sample and alternative predictors defined [17,39,43,44,45]. Indeed, so conspicuously successful have been these replications that the equation became known in the presidential studies literature as the “Simonton model” [43].^4^

However, the predictive relevance of Intellectual Brilliance was seriously challenged when Rubenzer and Faschingbauer devised their assessment of presidential Openness to Experience by surveying presidential biographers [29,30]. The researchers showed that Openness displayed significant correlations with presidential performance (*r*s = 0.25−0.32, [29]; using [37,41]). Although the zero-order correlations were lower than those found using Intellectual Brilliance, any direct comparison is somewhat misleading because the scores are not based on the same presidents. Where Intellectual Brilliance features scores for all 39 presidents up to Reagan, the Openness scores were missing for several presidents who were too obscure to attract a sufficient number of biographers (e.g., John Tyler, Zachary Taylor, Rutherford Hayes, Grover Cleveland, and William McKinley). Hence, a decision was made to attempt a head-to-head comparison that relied on imputation methods for estimating missing values. To provide more data for the imputed scores, Cox’s [7] four IQ estimates were added as well. Given that no president was missing a value across all three measures—Intellectual Brilliance, Openness, and IQ—it was then possible to obtain scores for all presidents from Washington to George W. Bush. The results were reported by Simonton ([3], Table 1). 

Because the focus of this article is the intelligence–performance relation, let us concentrate on the relative predictive utility of these three alternative indicators. In this case, presidential performance was assessed by a 12-item composite that incorporated the ratings from all major expert surveys (i.e., the measure with the 0.99 coefficient alpha mentioned earlier). Then the following two points must be emphasized [3]: First, Intellectual Brilliance claims a far higher correlation with presidential performance (*r* = 0.56) than does either Openness to Experience (*r* = 0.34) or any of the four Cox IQ estimates (*r*s = 0.31−0.34). Second, only Intellectual Brilliance makes a statistically significant contribution to the Simonton six-predictor model (β = 0.29, *p* < 0.01; which is actually slightly higher than the 0.26 as first found in [2]). In contrast, if Intellectual Brilliance is replaced by either Openness or any of the Cox IQ estimates, their predictive utility shrinks to a nonsignificant effect (β = 0.19, *p* > 0.05). In other words, without Intellectual Brilliance in our inventory of available predictors, we would be obliged to fall back on a five-predictor equation containing just the factors of years in office, war years, assassination, scandals, and war hero status (cf. [33]). The total amount of variance explained would be correspondingly reduced. To illustrate, omitting Intellectual Brilliance from the equation predicting the Murray and Blessing [41] ratings lowers the percentage of variance accounted for from 82% to 77% (cf. [3,33]). That lost increment of 5% is not trivial.^5^

If Intellectual Brilliance cannot be equated with either IQ or Openness, we might ask how it compares with Sternberg’s [49] concept of Successful Intelligence, which he discusses in this special issue. Some congruence is apparent, but not enough to make the two concepts equivalent. In the first place, analytical intelligence plays a much bigger and more explicit role in Successful Intelligence, whereas Intellectual Brilliance contains only the single generic descriptor of Intelligent, which might even be taken to encompass a broader intelligence, such as Gardner’s [50] construct of interpersonal intelligence. Second, although both Intellectual Brilliance and Successful Intelligence include creativity as a component, creativity plays a much bigger role in the latter than in the former. Not only does Intellectual Brilliance omit most of the adjectives making up the ACL’s Creative Personality Scale (e.g., Confident, Egotistical, Individualistic, Informal, Original, Resourceful, Sexy, and Snobbish; [51]), but Simonton [17] was also able to extract a measure of Creative Style that could be empirically differentiated from Intellectual Brilliance. Third, although Wise is included for both Intellectual Brilliance and Successful Intelligence, common sense is not; nor is practicality, even though Practical is included among the descriptors in the ACL [3]. Maranell’s [21] survey measure of Practicality is even negatively correlated with Intellectual Brilliance [2]. Finally, Intellectual Brilliance contains traits that do not seem to have any counterpart in Successful Intelligence, such as Sophisticated and Idealistic. In short, however essential Successful Intelligence may be for success in general, Intellectual Brilliance is more specifically tailored to what it takes to attain presidential greatness.^6^

## 5. Final Inferences

It should be clear by now than all internet debates about the IQs of United States presidents are terribly misplaced. It doesn’t really matter much whether George W. Bush has an IQ of 91, or Donald Trump an IQ of 156. What we all should be debating is the Intellectual Brilliance of the person occupying the White House. Although this factor includes intelligence among its components, that trait is only one among a dozen, and even then features a rather middling factor loading. We also have to ask such questions as: How much curiosity and breadth of interests does the person display? Is the leader sophisticated, even complicated, rather than dull or commonplace? How inventive, artistic, or insightful are they? And what about the leader’s wisdom and idealism? Even if in varying degrees, these are evidently the qualities that are most strongly associated with exceptional presidential performance, an inclusive criterion that incorporates such specific leader assets as Strength, Prestige, Activity, Accomplishments, and Political Skill. This identifies the leadership that is most likely to solve the problems of today and anticipate the problems of tomorrow. Or, at least, these are the leaders who are most likely to be open to those who offer innovative solutions to those problems. We witness today the consequences of an American presidency that spurns the best that science has to offer with respect to the biggest problems of our time. 

Such were the implications that should have been drawn from Simonton’s [3] systematic comparison of presidential Intellectual Brilliance, Openness, and IQ. Yet, everyone who downloaded the article seems to have become fixated on the IQ scores, ignoring the far more crucial scores of Intellectual Brilliance. To provide a concrete example, many on the internet observed that J. Q. Adams ended up with the highest IQ estimate: somewhere between 165 and 175. In contrast, one of the few bona fide geniuses ever to serve as president, Thomas Jefferson, only scored somewhere between 145 and 160, and thus their IQ estimates didn’t even overlap. If their respective records of early intellectual development are scrutinized as closely as Cox’s [7] raters did, this disparity makes perfect sense. Adams was clearly the more cognitively precocious. Only when we view the column containing the Intellectual Brilliance scores do we spot a dramatic contrast. Where J. Q. Adams was only a little more than one standard deviation above the presidential mean, Jefferson was more than three standard deviations above that same mean! Indeed, Jefferson displayed more Intellectual Brilliance than any president who ever served in that office! Then, who was the greater president? Jefferson by far! Where J. Q. Adams is most often rated as an only slightly above-average chief executive, Jefferson is usually placed in the top four—right below the triumvirate of Washington, Lincoln, and Franklin Roosevelt. Indeed, Jefferson owns the visage right beside Washington on Mount Rushmore. The only misfortune in Jefferson’s posthumous reputation was his placement on the ill-fated $2 bill! So, please, anyone who wants to make the presidency great again has to know what really to look for. 

The obvious next question is whether the voters in presidential elections can be encouraged or trained to cast their ballots more wisely. The answer is far from easy because a host of extraneous factors impinge on the ideal “rational voter” (cf. [53,54]). For one thing, voters are often more strongly influenced by emotion rather than cognition, relying excessively on spontaneous emotional reactions that may even be governed by primitive processes that hark back to the early phases of human evolution [55]. Worse yet, voter behavior is heavily shaped by situational factors that have nothing to do with the specific candidates running for the nation’s highest office [1]. For example, several such contextual factors would predict that the person inaugurated as U.S. president in 2017 would be affiliated with the Republican rather than Democratic Party, regardless of who the nominees happened to be. After all, the Democrats had occupied the White House for two consecutive terms, and had already lost control of both branches of Congress as well as most state governorships and legislatures. Warren G. Harding, who is often considered the worse chief executive ever, was elected under somewhat similar circumstances. Yet, he was the president whose Intellectual Brilliance fell two standard deviations below the mean. Absolutely nobody scored lower! 

In any event, if Intellectual Brilliance rather than IQ or Openness to Experience represents the primary personal factor behind presidential performance, then can that distinctive configuration of traits generalize to other forms of political leadership, or even to leadership positions in general? 

On the one hand, it could be that this specific factor is distinctive to the U.S. presidency, and that other positions, such as British Prime Minister or CEO of Fortune 500 companies, require a somewhat different mix of characteristics; albeit some subset may incorporate intelligence, creativity, openness, and perhaps wisdom or idealism. If so, such complications would render the solution to the world’s urgent problems all the more difficult. In the absence of a one-size-fits-all profile for effective leadership, each political system must identify its idiosyncratic pattern of optimal traits—and then put somebody in office who exemplifies those traits. 

On the other hand, it might hold that Intellectual Brilliance can serve as a much broader basis for predicting leader performance. Precisely the same contributing traits may be involved, but with only slight alterations in the weights they are assigned. It must be stressed that all traits defining this factor represent characteristics applicable to the general population. That inclusive applicability was guaranteed when the ACL was originally standardized. Because everybody can vary on these traits, everyone can vary in estimated Intellectual Brilliance as well. That variation might then predict performance in a wider range of domains besides leadership. In partial support of this conjecture are the results of Simonton’s [25] secondary analysis of the at-a-distance assessments that Thorndike [9] posthumously published for 91 eminent creators and leaders. This analysis revealed a factor defined by sensitiveness; intelligence; and a liking for art, music, beauty, words, reading, and things. This trait configuration seems to roughly approximate Intellectual Brilliance. Indeed, Knapp [56] had earlier labelled this complex factor as an “intellectual sensitivity” rather than “intelligence” dimension. Yet, this very same factor emerged as the prime predictor of achieved eminence across all domains, from artists, scientists, and inventors to politicians, military figures, and entrepreneurs. 

Without doubt, more research is required before the impact of Intellectual Brilliance can be fully evaluated. Yet one conclusion is safe to make right now: just picking heads of state according to hypothetical scores on an IQ test will not accomplish anything useful. That inference is powerful because the solutions to the world’s problems very often must be implemented by its leaders. To offer a specific example, consider the following recent case: A virtually universal consensus exists among scientists that the earth is rapidly warming, and that human production of CO_2_ is the main causal agent. So persuasive is the case that the majority of the world’s nations were willing to sign the 2016 Paris Agreement dedicated to the reduction of greenhouse gases. Even so, it only took one leader, the president of the most powerful (but also most polluting) nation on the planet, to decide to withdraw his country from this epochal accord—under the utterly unjustified belief that climate change was a big hoax!

## Footnotes

1 Technically speaking, the same labor-intensive methods could be extended to former president Barack Obama, once the requisite biographical data become available (cf. [2]). In contrast, these methods cannot be fully applied to the current incumbent until after he completes his term in office. Nonetheless, I leave it as an exercise for the reader to estimate how the current incumbent might rate on the descriptors defining the Intellectual Brilliance factor.

2 The same biographical personality profiles have been used in later research to extract additional characteristics, such as creativity, charisma, narcissism, and proactivity [17,18,19].

3 It should be evident that Intellectual Brilliance has little, if any, conceptual overlap with Emotional Intelligence, a popular construct that has often been associated with effective leadership [23,24]. Most notably, the former concerns cognition, the latter emotion. If each defining trait is scrutinized one by one, it is difficult to discern any connection with affect, as defined in Emotional Intelligence.

4 These successive replications involved more than just applying the equation to an enlarged sample upon each new survey. The equation would also test whether other potential predictors should be added, or even replace the predictors in the original equation. Many individual and situational variables exhibit zero-order correlations with presidential performance measures, but without adding any increment to the explained variance once the other six variables are already in the equation. That means that the correlation represents some combination of indirect and/or spurious relationships (see [46] for further discussion).

5 Although some have suggested that leadership might be a curvilinear inverted-U function of intelligence, the relation between Intellectual Brilliance and presidential greatness is strictly positive linear (cf. [47,48]). Nonetheless, an adverse effect is found in another location: Intellectual Brilliance tends to be negatively correlated with a candidate’s margin of victory in the popular vote (*r* = −0.36 [2]). The 2016 presidential election just might be an exception to this statistical relation.

6 Because Intellectual Brilliance may put more stress on personality in comparison with Successful Intelligence, it might be more analogous to the concept of the “intelligent personality” and academic performance, but with that cluster of dispositional traits focused on presidential leadership (cf. [52]). That is where this performance predictor has greater affinity with Openness as well.

## References

[1] Simonton D.K., Rumsey M.G. (2012). Presidential leadership: Performance criteria and their predictors. The Oxford Handbook of Leadership.

[2] Simonton D.K. (1986). Presidential personality: Biographical use of the Gough Adjective Check List. J. Pers. Soc. Psychol..

[3] Simonton D.K. (2006). Presidential IQ, Openness, Intellectual Brilliance, and leadership: Estimates and correlations for 42 US chief executives. Polit. Psychol..

[4] Simonton D.K. (2009). The “other IQ”: Historiometric assessments of intelligence and related constructs. Rev. Gen. Psychol..

[5] Song A.V., Simonton D.K., Robins R.W., Fraley R.C., Krueger R.F. (2007). Personality assessment at a distance: Quantitative methods. Handbook of Research Methods in Personality Psychology.

[6] Woods F.A. (1906). Mental and Moral Heredity in Royalty.

[7] Cox C. (1926). The Early Mental Traits of Three Hundred Geniuses.

[8] Thorndike E.L. (1936). The relation between intellect and morality in rulers. Am. J. Sociol..

[9] Thorndike E.L. (1950). Traits of personality and their intercorrelations as shown in biographies. J. Educ. Psychol..

[10] Simonton D.K. (1983). Intergenerational transfer of individual differences in hereditary monarchs: Genetic, role-modeling, cohort, or sociocultural effects?. J. Pers. Soc. Psychol..

[11] Terman L.M. (1925–1959). Genetic Studies of Genius.

[12] Robinson A., Simonton D.K., Robinson A., Jolly J.L. (2014). Catharine Morris Cox Miles and the lives of others (1890–1984). A Century of Contributions to Gifted Education: Illuminating Lives.

[13] Simonton D.K. (2016). Reverse engineering genius: Historiometric studies of exceptional talent. Ann. N. Y. Acad. Sci..

[14] Simonton D.K. (2008). Childhood giftedness and adulthood genius: A historiometric analysis of 291 eminent African Americans. Gift. Child Q..

[15] Armbruster M.E. (1982). The Presidents of the United States and Their Administrations from Washington to Reagan.

[16] Gough H.G., Heilbrun A.B. (1965). The Adjective Check List Manual.

[17] Simonton D.K. (1988). Presidential style: Personality, biography, and performance. J. Pers. Soc. Psychol..

[18] Deluga R.J. (1997). Relationship among American presidential charismatic leadership, narcissism, and related performance. Leadersh. Q..

[19] Deluga R.J. (1998). American presidential proactivity, charismatic leadership, and rated performance. Leadersh. Q..

[20] Emrich C.G., Brower H.H., Feldman J.M., Garland H. (2001). Images in words: Presidential rhetoric, charisma, and greatness. Adm. Sci. Q..

[21] Maranell G.M. (1970). The evaluation of presidents: An extension of the Schlesinger polls. J. Am. Hist..

[22] Simonton D.K. (1981). Presidential greatness and performance: Can we predict leadership in the White House?. J. Pers..

[23] Goldman D. (1995). Emotional Intelligence: Why It Can Matter More Than IQ.

[24] Salovey P., Mayer J.D. (1990). Emotional intelligence. Imagin. Cogn. Pers..

[25] Simonton D.K. (1991). Personality correlates of exceptional personal influence: A note on Thorndike’s (1950) creators and leaders. Creat. Res. J..

[26] John O.P., Pervin L.A. (1990). The “Big Five” factor taxonomy: Dimensions of personality in the natural language and in questionnaires. Handbook of Personality Theory and Research.

[27] McCrae R.R., Greenberg D.M., Simonton D.K. (2014). Openness to experience. The Wiley Handbook of Genius.

[28] Ilies R., Gerhardt M.W., Le H. (2004). Individual differences in leadership emergence: Integrating meta-analytic findings and behavioral genetics estimates. Int. J. Sel. Assess..

[29] Rubenzer S.J., Faschingbauer T.R., Ones D.S. (2000). Assessing the U.S. presidents using the revised NEO Personality Inventory. Assessment.

[30] Rubenzer S.J., Faschingbauer T.R. (2004). Personality, Character, & Leadership in the White House: Psychologists Assess the Presidents.

[31] Simonton D.K. (2004). Does character count in the Oval Office?. PsycCRITIQUES.

[32] Schlesinger A.M. (1948). Historians rate the U.S. presidents. Life.

[33] Simonton D.K. (1986). Presidential greatness: The historical consensus and its psychological significance. Polit. Psychol..

[34] Balz J. (2010). Ready to lead on day one: Predicting presidential greatness from political experience. PS Polit. Sci. Polit..

[35] Curry J.L., Morris I.L. (2010). Explaining presidential greatness: The roles of peace and prosperity?. Pres. Stud. Q..

[36] Wendt H.W., Light P.C. (1976). Measuring “greatness” in American presidents: Model case for international research on political leadership?. Eur. J. Soc. Psychol..

[37] Ridings W.J., McIver S.B. (1997). Rating the Presidents: A Ranking of U.S. Leaders, from the Great and Honorable to the Dishonest and Incompetent.

[38] Simonton D.K. (1991). Latent-variable models of posthumous reputation: A quest for Galton’s *G*. J. Pers. Soc. Psychol..

[39] Simonton D.K. (2001). Predicting presidential greatness: Equation replication on recent survey results. J. Soc. Psychol..

[40] Simonton D.K. (1984). Leaders as eponyms: Individual and situational determinants of monarchal eminence. J. Pers..

[41] Murray R.K., Blessing T.H. (1983). The presidential performance study: A progress report. J. Am. Hist..

[42] Simonton D.K. (1987). Presidential inflexibility and veto behavior: Two individual-situational interactions. J. Pers..

[43] Cohen J.E. (2003). The polls: Presidential greatness as seen in the mass public: An extension and application of the Simonton model. Pres. Stud. Q..

[44] Simonton D.K. (1991). Predicting presidential greatness: An alternative to the Kenney and Rice Contextual Index. Pres. Stud. Q..

[45] Simonton D.K. (2002). Intelligence and presidential greatness: Equation replication using updated IQ estimates. Advant. Psychol. Res..

[46] Simonton D.K., Hoyt C., Goethals G.R., Forsyth D. (2008). Presidential greatness and its socio-psychological significance: Individual or situation? Performance or attribution?. Leadership at the Crossroads: Psychology and Leadership.

[47] Antonakis J., House R.J., Simonton D.K. (2017). Can super smart leaders suffer too much from a good thing? The curvilinear effect of intelligence on perceived leadership behavior. J. Appl. Psychol..

[48] Simonton D.K. (1985). Intelligence and personal influence in groups: Four nonlinear models. Psychol. Rev..

[49] Sternberg R.J. (2018). Speculations on the role of successful intelligence in solving contemporary world problems. J. Intell..

[50] Gardner H. (1983). Frames of Mind: A Theory of Multiple Intelligences.

[51] Gough H.G. (1979). A Creative Personality Scale for the Adjective Check List. J. Pers. Soc. Psychol..

[52] Chamorro-Premuzic T., Furnham A. (2006). Intellectual competence and intelligent personality: A third way in differential psychology. Rev. Gen. Psychol..

[53] Simonton D.K. (1993). Further Details on VOTER HELPER^TM^ 1.0: A response to the editor’s comments. Polit. Psychol..

[54] Simonton D.K. (1993). Putting the best leaders in the White House: Personality, policy, and performance. Polit. Psychol..

[55] Shenkman R. (2016). Political Animals: How Our Stone-Age Brain Gets in the Way of Smart Politics.

[56] Knapp R.H. (1962). A factor analysis of Thorndike’s ratings of eminent men. J. Soc. Psychol..

